# Gemcitabine and vinorelbine followed by docetaxel in patients with advanced non-small-cell lung cancer: a multi-institutional phase II trial of nonplatinum sequential triplet combination chemotherapy (JMTO LC00-02)

**DOI:** 10.1038/sj.bjc.6600723

**Published:** 2003-02-10

**Authors:** S Hosoe, K Komuta, K Shibata, H Harada, Y Iwamoto, Y Ohsaki, T Morioka, H Origasa, M Fukushima, K Furuse, M Kawahara

**Affiliations:** 1 Department of Internal Medicine, National Kinki-Central Hospital for Chest Diseases, 1180 Nagasone-cho, Sakai-city, Osaka 591-8555, Japan; 2 Japan-Multinational Trial Organization (JMTO), 54 Shogoin Kawara-cho, Sakyo-ku, Kyoto, 606-8507, Japan; 3 Division of Biostatistics, Faculty of Medicine, Toyama Medical and Pharmaceutical University, 2630 Sugitani, Toyama 930-0194, Japan; 4 Department of Pharmacoepidemiology, Graduate School of Medicine, 54 Shogoin Kawara-cho, Sakyo-ku, Kyoto, 606-8507, Kyoto University, Kyoto, Japan

**Keywords:** advanced non-small-cell lung cancer, nonplatinum regimen, sequential-triplet chemotherapy

## Abstract

To evaluate the efficacy and toxicity of the sequential nonplatinum combination chemotherapy consisting of gemcitabine (GEM) and vinorelbine (VNR) followed by docetaxel (DOC) in patients with advanced non-small-cell lung cancer (NSCLC), we conducted the multiinstitutional phase II study. A total of 44 chemotherapy-naive patients with advanced NSCLC were treated with GEM 1000 mg m^−2^ and VNR 25 mg m^−2^ intravenously on days 1 and 8 every 3 weeks for three cycles. DOC 60 mg m^−2^ was then administrated intravenously at 3-week intervals for three cycles. Patients were evaluated for response and toxicity with each cycle of the treatment. The major objective response rate was 47.7% (95% confidence interval (CI), 33.8–62.1%). Median survival time (MST) was 15.7 months and 1-year survival rate was 59%. In the GEM/VNR cycle, grade 3/4 neutropenia occurred in 36.3%, grade 3/4 anaemia in two patients (4.5%) and grade 3 thrombocytopenia in one patient (2.3%). Grade 3 pneumonitis occurred in two patients (4.5%) in GEM/VNR cycles. In the DOC cycles, grade 3/4 neutropenia occurred in 39.4% but no patient experienced grade 3/4 anaemia or thrombocytopenia. Of the 44 eligible patients, 33 patients completed three cycles of GEM/VNR and 22 patients completed six cycles of planned chemotherapy (three cycles of GEM/VNR followed by three cycles of DOC). The sequential triplet nonplatinum chemotherapy consisted of GEM/VNR followed by DOC, and was very active and well tolerated. This study forms the basis for an ongoing phase III trial that compares this nonplatinum triplet and standard platinum doublet combination (carboplatin/paclitaxel).

Lung cancer represents a major cause of cancer death in many countries and approximately 80% of all these patients are categorised as non-small-cell lung cancer (NSCLC) (Fry *et al*, 1996). Cisplatin (CDDP)-containing chemotherapy has been shown to have certain benefits in the survival of patients with advanced NSCLC (NSCLC Cooperative Group, 1995). However, combination chemotherapy containing CDDP has significant toxicities, including severe nausea, vomiting and renal toxicity requiring adequate hydration, which increases the difficulty in the treatment of the elderly or outpatients. In previous randomised studies, CDDP-containing combinations have proved more toxic than those without cisplatin (Luedke *et al*, 1990; Gridelli *et al*, 1996; Georgoulias *et al*, 2001). Carboplatin is a platinum without the toxic disadvantages of CDDP; however, this agent still causes nausea, vomiting and myelosuppression. New active agents such as taxanes, gemcitabine (GEM), vinorelbine (VNR) and topoisomerase-I inhibitors have been tested in NSCLC with encouraging results, and several nonplatinum combinations have been developed. Among the new drugs, GEM/VNR combination is noteworthy because of their demonstrated activity and particularly their good toxicity profile. This combination can also be used for elderly or unfit patients, because of its low toxicities (Feliu *et al*, 1999; Isokangas *et al*, 1999; Bajetta *et al*, 2000; Beretta *et al*, 2000; Gridelli *et al*, 2000). In addition, it has been shown that a single treatment of docetaxel (DOC) is active for NSCLC, especially as a second-line treatment (Fossella *et al*, 2000; Shephard *et al*, 2000). This led to the hypothesis that DOC may be effective for the resistant clones against first-line chemotherapy. A small amount of residual resistant clones can be eradicated by sequential administration of DOC before these clones grow and relapse. Some studies showed that a combined chemotherapy of three agents (triplet) may prove to be superior to that of two agents (doublet) (Comella *et al*, 2000). However, concurrent administration of triplet combination requires the reduction of dose because of toxicities, and subsequently may cause the reduction of effectiveness. Theoretical modelling of Norton–Simon (1986) clearly indicates that cell kill can be substantially increased by the sequential use of cytotoxic regimens. However, the Norton–Simon hypothesis has only been validated in a minority of cancer treatment strategies. The dose of GEM/VNR combination was determined according to several phase II trials, especially the Italian study that compared three different doses (Gridelli *et al*, 2000). A 60 mg m^−2^ of DOC was the recommended dose in a Japanese phase I study. Japanese phase II studies were carried out at this dose and were not inferior when compared with several studies with more than 70 mg m^−2^ of DOC. Regarding the number of cycles, an average of 3–4 cycles per patient were given in two phase III studies of second-line DOC (Fossella *et al*, 2000; Shephard *et al*, 2000). It has been reported that no additional benefits were observed by continuing chemotherapy beyond three cycles (Smith *et al*, 2001). Based on this collective background, we conducted this phase II trial of a sequential nonplatinum triplet combination consisting of three cycles of GEM (100 mg m^−2^) and VNR (25 mg m^−2^) followed by three cycles of DOC (60 mg m^−2^).

## PATIENTS AND METHODS

### Patient eligibility

Chemotherapy-naïve patients with Eastern Cooperative Oncology Group (ECOG) performance status (PS) 0–1, over 18 years old, with stage IV and IIIB (with malignant pleural effusion and/or pulmonary nodule(s) that the same lobe of the primary lesion) NSCLC were eligible. The upper limit of age was not defined. Patients had unidimensionally measurable disease. Exclusion criteria were the presence of apparent interstitial pneumonitis, massive pleural effusion requiring thoracenthesis, uncontrollable diabetes mellitus, heart diseases, history of another cancer (excluding nonmelanomatous skin cancer and *in situ* cervical cancer), reduced bone marrow, pulmonary, renal or hepatic function. Stage IIIB patients with pulmonary nodule(s) at the same lobe of the primary lesion were ineligible if they could be considered as an indication of radiation therapy or operation. CNS metastases were not considered as an exclusion criterion if asymptomatic. All patients gave written informed consent. The study protocol was approved by the ethical committees of Japan Multinational Trial Organization (JMTO) and the participating institutions.

### Treatment plan

Within 7 days before entry in the study, all patients underwent a complete medical history, physical examination, urine, haematologic and biomedical testing. Within 4 weeks, clinically indicated scans, including computed tomography of the chest, abdomen (or abdominal ultrasound), brain (or magnetic resonance imaging of the brain) and radionuclide bone scans, electrocardiogram, arterial blood gas and pulmonary function tests were performed. Patients received GEM 1000 mg m^−2^ and VNR 25 mg m^−2^ on days 1 and 8 every 21 days for three cycles. Single-agent DOC 60 mg m^−2^ was then given on day 1 every 21 days for three cycles. Premedications such as antiemetic agents or corticosteroids were given at the investigator's discretion. Prophylactic granulocyte-colony stimulating factor (G-CSF) was not allowed at any treatment cycle. During the DOC cycle, all patients received 8 mg of dexamethasone just before the administration of DOC. Complete blood cell count was checked on the day of each planned treatment. During the GEM/VNR cycle, a liver function test (AST and ALT) was also administrated. If WBC count was below 3000 mm^−3^, platelet count was below 75 000 mm^−3^, or AST/ALT was over 100 IU l^−1^ on day 1 of each cycle, GEM/VNR administration was delayed by a week. If WBC count was below 2000 mm^−3^, platelet count was below 50 000 mm^−3^ or AST/ALT was over 100 IU l^−1^, administration of GEM/VNR on day 8 was discontinued. If WBC count was below 3000 mm^−3^ or platelet count was below 75 000 mm^−3^ at day 1 of the cycle, DOC administration was delayed by a week. Toxicity evaluations were based on the National Cancer Institute's Common Toxicity Criteria (NCI-CTC), Version 2.0. Treatment dose was reduced to 80% of prior treatment dose if there were grade 4 leukocytopenia, neutropenia or platelet counts below 20 000 mm^−3^, other unacceptable toxicities including grade 3 neutropenic fever or grade 3 or more nonhaematological toxicities other than nausea, vomiting, fatigue or alopecia, during the preceding treatment cycle. A full dose of DOC was administrated on day 1 of the first DOC cycle, even if elevated toxicities were observed in the prior GEM/VNR cycles. The dose of DOC was reduced to 80% only when the above toxicities were observed by prior administration of DOC. Patients went off-study with a treatment delay of greater than 2 weeks or with progressive disease. Protocol treatment was also discontinued when patients had grade 2 pneumonitis or other severe toxicities that made it difficult to continue the protocol treatment. Patients were monitored and evaluated weekly for toxicities and monthly for response. Response evaluation criteria in solid tumor (RECIST) (Therasse *et al*, 2000) was used for the evaluation of response. An extramural review of all treated patients was performed for the response evaluation.

### Statistical methods

In accordance with the optimal two-stage phase II design (Simon, 1989), the treatment programme was designed to reject a response rate of less than 20% (*p*0) and provide a statistical power of 85% in assessing the activity of the regimen as 40% (*p*1) (*p*1−*p*0=20%) with an alpha error of less than 0.05 and a beta error of less than 0.10. Therefore, 40 patients were required: 22 for the first step and 18 for the second. Survival was calculated from the day of registration to the day of death using the Kaplan–Meier method (1958).

## RESULTS

A total of 45 patients were enrolled from 17 participating institutions between April and November 2000. After registration, one patient was excluded because he was proved to be stage 1 and had an operation. Patient characteristics are listed in Table 1

**Table 1:** Patient characteristics

**Characteristics**	**No.**	**%**
*Total*	44	100
*Sex*		
Male	28	63.6
Female	16	36.4
*Age (years)*		
Median (range)	63	(44–81)
<70	36	81.8
⩾70	8	18.2
*ECOG performance status*		
0	9	20.5
1	35	79.5
*Histology*		
Adenocarcinoma	29	65.9
Squamous cell	10	22.7
Large cell	5	11.4
*Stage*		
IIIB	9	20.5
IV	35	79.5
*No. of distant metastases*		
0	9	20.5
1	24	54.5
2	7	15.9
3	3	6.8
⩾4	1	2.3

ECOG=Eastern Cooperative Oncology Group.

. Of the 44 eligible patients, there were 28 men and 16 women, with a median age of 63 years (range, 41–81 years). The majority of the patients had stage IV disease and ECOG PS of 1. The results for response rate, toxicity profile and survival were analysed on September 2001. As shown in Table 2

**Table 2:** Reasons and timing for treatment discontinuation

	**GEM/VNR**			**DOC**			
**Cycle no.**	**1**	**2**	**3**	**4**	**5**	**6**	**Total**
Progressive disease	0	3	4	2	0	1	10
Adverse event	0	1	2	0	1	2	6
Patient's refusal	0	1	0	2	2	1	6
							
**Figure 1:**
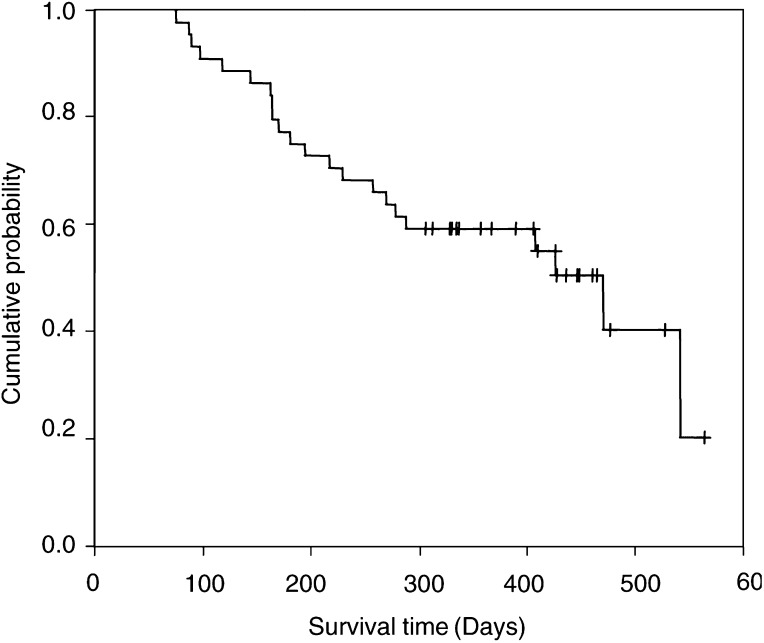
Kaplan–Meier survival curve is shown. With a mean follow-up of 374.5 days, the predicted median survival time is 15.7 months and the 1-year survival rate is 59%.

							22

, 33 (75%) out of 44 patients received three cycles of GEM/VNR, and during the DOC cycle, 22 (76%) out of 29 patients completed three cycles of DOC. Of 44 patients, 11 patients went off the protocol during GEM/VNR cycle (seven patients had progressive disease, two patients had pneumonitis, one had treatment delay more than 2 weeks because of grade 2 hepatic dysfunction and one patient withdrew informed consent). Of 33 patients who completed three cycles of GEM/VNR, four patients went off the protocol just before the DOC cycle (two patients had progressive disease and two patients withdrew informed consent). Accordingly, 29 out of 44 registered patients (66%) went to DOC cycle. During DOC cycle, seven out of 29 patients went off protocol (three patients withdrew informed consent, one had progressive disease, two had treatment delay because of myelosuppression, one had grade 2 pneumonitis). Therefore, 22 out of 44 (50%) patients completed six cycles of chemotherapy. The average cycle number of chemotherapy was 4.5 cycles per patient. As a whole, there were no complete response (CR), 21 partial response (PR), 17 stable disease (SD) and six progressive disease (PD). The best overall response rate was 47.7% with 95% CI: 31.8–61.4%. MST was 15.7 months (=471 days, 95% CI: 270 days to not yet reached) with a mean follow-up time of 374.5 days. The 1-year survival rate was 59% and the Kaplan–Meier survival curve is displayed in Figure 1

. The survival data were analysed in October 2001, at which time, 22 patients had died and 22 were alive. The toxicity profile is shown in Table 3

**Table 3:** Frequency of toxicities (worst toxicities per patient) during the first three cycles (GEM/VNR) and the latter three cycles (DOC)

	**GEM/VNR (*n*=44)**		**DOC (*n*=33)**	
	**Grade3 (%)**	**Grade4 (%)**	**Grade3 (%)**	**Grade4 (%)**
*Haematologic*				
Neutrophils	20.4	15.9	15.2	24.2
Haemoglobin	2.3	2.3	0	0
Platelets	2.3	0	0	0
				
*Nonhaematologic*				
Nausea, vomiting	2.3	0	3.0	0
Febrile neutropenia	2.3	0	0	0
Pneumonitis	4.5	0	0	0
Fatigue	4.5	0	0	0
Hepatic dysfunction	2.3	0	0	0
Hyponatremia	2.3	0	0	0
Hypopotassemia	2.3	0	0	0
Haematuria	2.3	0	0	0

. There were no treatment-related deaths. Grade 3/4 neutropenia was seen in 36.3 and 39.4% in GEM/VNR and DOC cycle, respectively. There were only two patients (4.5%) and one patient (2.3%) who experienced grade 3/4 anaemia and thrombocytopenia, respectively. In nonhaematologic toxicities, two patients (4.5%) had grade 3 pneumonitis during the GEM/VNR cycle. Other toxicities were infrequent and generally acceptable. A mild elevation of AST/ALT was seen occasionally in the GEM/VNR cycle, but except for one patient (grade 3) most occurrences were in grade 1/2 and all of them recovered without treatment. There was no neurological toxicity corresponding to grade 3/4, but six patients experienced grade 1 during the GEM/VNR cycle (data not shown).

## DISCUSSION

The recent development of new anticancer drugs, which have almost equal activity against NSCLC but are less toxic compared to CDDP, provides alternatives to CDDP (Georgoulias *et al*, 2001). In particular, a GEM/VNR combination was very well tolerated with high activity (Feliu *et al*, 1999; Isokangas *et al*, 1999; Bajetta *et al*, 2000; Beretta *et al*, 2000; Gridelli *et al*, 2000). The Spanish Lung Cancer Group (Alberola *et al*, 2001) reported on a randomised comparison of a CDDP-based three-drug combination (CDDP/GEM/VNR) *vs* non-CDDP sequential doublets (GEM/VNR followed by ifosphamide/VNR) *vs* CDDP/GEM (reference regimen). There were no survival differences among the three arms, and it was concluded that the triplet combination including CDDP showed no advantage since the toxicity profile of this arm was worst. This result is contrary to that of an Italian phase III study by Comella *et al* (2000). The best toxicity profile in the Spanish study was seen in the arm without CDDP (GEM/VNR followed by ifosphamide/VNR), a sequential combination. There is still some anxiety for the combination without CDDP whether it may lose its power of chemotherapy; however, there have been some encouraging reports without CDDP for NSCLC. Pectasides *et al* (1999) reported a triplet combination of carboplatin, DOC and GEM for NSCLC with a response rate of 46.5% and MST of 13.5 months. Another report by Miller *et al* (2000) showed a response rate of 51% and a 1-year survival of 60% by DOC and VNR. Both these phase II studies showed high response rates and good survival, but febrile neutropenia was seen in 13–14% of the patients even though both studies used prophylactic G-CSF and the latter study used prophylactic ciprofloxacin, as well. In the present study, only one patient (2%) developed febrile neutropenia, but we did not use prophylactic G-CSF or antibiotics. Furthermore, in contrast to conventional platinum-based chemotherapy in which most patients experience nausea, vomiting and fatigue, only a limited number of patients had these toxicities in this nonplatinum triplet regimen. Although we did not evaluate the quality of life (QoL), this regimen could in fact prove superior to conventional platinum-based regimens in terms of QoL.

Recently, DOC was shown to be active as a second-line chemotherapy (Fossella *et al*, 2000; Shephard *et al*, 2000). On the assumption that the tumour is composed of subpopulations of cells with differing patterns of resistance and growth kinetics, DOC may be effective against the tumour population of refractory but slower-growing cells. Therefore, sequential administration of DOC following other agents as a first-line may strike the refractory population before they grow and become apparent. Another rationale for exploring sequential therapy is optimising dose delivery. The concept for the sequential administration of full-dose chemotherapeutic agents having different mechanisms of action is supported by the Norton–Simon hypothesis (1986). Manegold *et al* (2001) reported on a sequential administration of single-agent GEM and DOC. Although it was a randomised phase II study, they reported that a 4 weekly GEM–DOC sequence was better than a DOC–GEM sequence, in terms of survival. However, sequential but monotherapy may not be viewed positively since the response rate was 5–11% and the 1-year survival was 19–30% in their study. Recently, Edelman *et al* (2001a) reported the results of phase II study for sequential combination chemotherapy consisting of carboplatin and GEM followed by paclitaxel showed modest activity (overall response: 31%, median survival time: 9.5 months). Based on the results of this study, Southwest Oncology Group (SWOG) has completed a randomised phase II trial of two sequential combinations (CDDP/VNR followed by DOC *vs* carboplatin/GEM followed by paclitaxel) and reported that both arms had comparable activity (response rate: 21 and 28%, 1-year survival: 32 and 31%) (Edelman *et al*, 2001b). The concept of this SWOG study which involved a doublet followed by taxane, is similar to that of the present study. However, both arms of the SWOG study included platinum in the front line, so that the toxicity profile was comparatively worse. In the present study, 21 out of 44 patients showed PR. In all, 17 patients showed PR in the GEM/VNR cycle and four patients in the DOC cycle (data not shown). Tumours in some patients began to decrease in size during the GEM/VNR cycles and lasted through the DOC cycles. It is possible to consider that not only GEM/VNR but also DOC was effective in these patients. One patient went off-study in the GEM/VNR cycle because of treatment delay caused by prolonged hepatotoxicity. This patient had a PR by the DOC administration. Another patient who went off-study in the GEM/VNR cycle because of disease progression had a PR by the DOC administration. Although the response in these two patients were evaluated as SD and PD, but not as PR because the response was seen when the patients were off-study, sequential administration of DOC seems to be also effective for patients with resistant, or unfit, to other chemotherapeutic agents.

The best overall response rate in this study was 47.7% (95% CI: 31.4–61.8%). Median survival time was 15.7 months and the 1-year survival rate was 59%. These results are encouraging and may be one of the highest reported for advanced NSCLC. It should be pointed out that this finding, especially survival data, might have been influenced by patient selection. In this study, patients' PS might be relatively better than other trials. Although 80% of the patients were at stage IV, 83% of them had less than two sites of distant metastasis (Table 1). Furthermore, only three of them had solitary brain metastasis (data not shown) that was relatively small in size and asymptomatic. None of them were treated with cranial irradiation. There were no treatment-related deaths. Toxicities were mild and acceptable, with approximately one-third of patients showing grade 3/4 neutropenia, and only one and two patients showing grade 3/4 thrombocytopenia and anaemia, respectively. Most nonhaematological toxicities were mild and infrequent. However, there were two patients who experienced grade 3 pneumonitis in the GEM/VNR cycle. They received corticosteroid treatment and recovered in a couple of months, but both patients went into best supportive care. Even though this toxicity may be a rare event, attention should be paid since this can be life threatening. In a phase II study of three different doses of GEM/VNR in Italy (Gridelli *et al*, 2000), a grade 3 pneumonitis was observed in dose level II but not in dose level III, suggesting that this adverse effect is not dose dependent. There were eight patients aged over 70 years in our study. In these eight elderly patients, five patients (62.5%) completed six cycles of chemotherapy, and the response rate was 62.5% (95% CI: 25–87.5%). Therefore, this regimen seems apt for including elderly patients because of its high response rate and low toxicities. The results of Multicentre Italian Lung Cancer in the Elderly Study (MILES) showed that combination chemotherapy (GEM/VNR) was not superior to monotherapy (VNR) for elderly NSCLC patients (Gridelli *et al*, 2001), contrary to the Southern Italy Cooperative Oncology Group (SICOG) results (Frasci *et al*, 2000). In their studies, the administration dose of chemotherapy was higher than ours, so the toxicity profile was worse. They included patients with PS 2. At the decision for the recommended dose for phase II trial, the maximum tolerated dose as determined by the phase I study was sometimes chosen because investigators usually believe that a higher dose of chemotherapy makes for better efficacy. In the Italian phase II study of three different doses of GEM/VNR (Gridelli *et al*, 2000), however, the higher dose caused higher toxicities but no significant additional efficacy. Two phase III trials of second-line DOC (Fossella *et al*, 2000; Shephard *et al*, 2000) also showed that the arm of 75 mg of DOC was significantly better than that of 100 mg, in terms of survival. Therefore, regimens with doses considered adequate but not at maximum dose levels of multiple agents may be sufficient for NSCLC patients, including the elderly. Recently, Baldini *et al* (2001) reported the activity and safety of a nonplatinum-based triplet in advanced NSCLC. They reported 52% of response rate and 46.5% of 1-year survival but concurrent administration of three agents made a worse toxicity profile.

In conclusion, the nonplatinum sequential triplet regimen consisting of GEM/VNR followed by DOC is very attractive because of its high activity and low toxicity profile. This seems suitable for the elderly and outpatients, as well. We want to remark that the present study is the first report of nonplatinum sequential triplet chemotherapy against NSCLC. This study serves as the basis for an ongoing randomised phase III trial (JMTO LC00-03) that compares this regimen with carboplatin/paclitaxel. The major aim of this phase III study is to determine whether nonplatinum sequential triplet is superior to platinum doublet. The results of this study may also answer the question of whether or not platinum is essential to front-line chemotherapy for advanced NSCLC. The study, JMTO LC00-03, was begun in April 2001, in collaboration with SWOG 0003 trial (carboplatin/paclitaxel±tirapazamine), using the same protocol for common control arm (carboplatin/paclitaxel). In addition, it may also prove interesting if some determination of the ethnic background of patients might influence the chemotherapeutic effect and/or adverse effects on standard combination chemotherapy for advanced NSCLC.

## APPENDIX

*Japan-Multinational Trial Organization (JMTO) conducted this study (JMTO LC00-02) with the cooperation of the following investigators*: S Hosoe and M Kawahara, National Kinki Central Hospital for Chest Diseases, Osaka; M Asai and K Komuta, Osaka Police Hospital, Osaka; H Harada, Yao-Tokusyukai Hospital, Osaka; K Shibata, Koseiren Takaoka Hospital, Toyama; Y Iwamoto, Hiroshima Municipal Hospital, Hiroshima; Y Ohsaki, Asahikawa Medical College, Asahikawa; Y Yoshimi, Kanazawa Municipal Hospital, Kanazawa; K Aoe, National Sanyo Hospital, Hiroshima; M Takenaka and Y Tanio, Osaka Prefectural Hospital, Osaka; H Yamamoto, Iizuka Hospital, Fukuoka; Y Fujita, National Dohoku Hospital, Asahikawa; Y Sasaki, Kosei-Nenkin Hospital, Osaka; K Noto, Toyama Central Hospital, Toyama; H Kawasaki, National Okinawa Hospital, Okinawa; T Isobe, Hiroshima University, Hiroshima; T Nasu, Wakayama-Rosai Hospital, Wakayama; I Kishiro, Dokkyo Medical College, Tochigi.
